# Grain boundary resistance to amorphization of nanocrystalline silicon carbide

**DOI:** 10.1038/srep16602

**Published:** 2015-11-12

**Authors:** Dong Chen, Fei Gao, Bo Liu

**Affiliations:** 1 Department of Physics and Electronics, Henan University, Kaifeng 475004, P. R. China; 2 Department of Nuclear Engineering and Radiological Sciences, University of Michigan, Ann Arbor, MI 48109, USA

## Abstract

Under the C displacement condition, we have used molecular dynamics simulation to examine the effects of grain boundaries (GBs) on the amorphization of nanocrystalline silicon carbide (nc-SiC) by point defect accumulation. The results show that the interstitials are preferentially absorbed and accumulated at GBs that provide the sinks for defect annihilation at low doses, but also driving force to initiate amorphization in the nc-SiC at higher doses. The majority of surviving defects are C interstitials, as either C-Si or C-C dumbbells. The concentration of defect clusters increases with increasing dose, and their distributions are mainly observed along the GBs. Especially these small clusters can subsequently coalesce and form amorphous domains at the GBs during the accumulation of carbon defects. A comparison between displacement amorphized nc-SiC and melt-quenched single crystal SiC shows the similar topological features. At a dose of 0.55 displacements per atom (dpa), the pair correlation function lacks long range order, demonstrating that the nc-SiC is fully amorphilized.

Nanocrystalline (nc) structures have a large volume fraction of disordered intergranular regions, which are believed to have significantly different responses to radiation resistances as compared to single-crystalline materials. Many efforts have been devoted to explore radiation tolerance^1^ and suggest that nanostructured materials could become one of possible pathways to enhance the radiation resistance^2^,^3^,^4^. Silicon carbide (SiC) possesses a number of outstanding physical and chemical properties that make it suitable for semiconductor, microelectronic and optoelectronic device application, and structural materials in fusion reactors. Ishimaru *et al.*^5^ examined structural changes in SiC containing nanolayers of (111) planar defects, under electron beam irradiation by *in situ* transmission electron microscopy (TEM), and revealed the origin of the radiation resistance. They found that irradiation-induced point defects migrate two-dimensionally within the nanolayers defined by the (111) planar defects, and proposed that the two-dimensional limitation of defect migration is the origin of high radiation tolerance of SiC containing high-densities of planar defects. However, Jiang *et al.*^6^ found no improvement in resistance to amorphization of nc-SiC for heavy ion bombardment at room temperature. Although several experiments have shown that nc structures have increased radiation resistance due to the high density of grain boundaries (GBs), the detailed mechanisms are still controversial and largely unknown.

In order to focus on the defect responsible for amorphization and evaluate the effects of GBs in the nanocrystalline-to-amorphous (nc-a) transformation, it is critical to examine if nc-SiC can be amorphized by inducing displacements and specify the dose required for the nc-a transformation at the first step. In this report, we find that nc-SiC can be amorphized exclusively by carbon (C) displacements at a dose of 0.55 displacement per atom (dpa). The results show that the formation and subsequent coalescence of defect clusters at the GBs to become amorphous domains plays an important role in irradiation-induced amorphization of nc-SiC.

## Results

**Figure 1:**
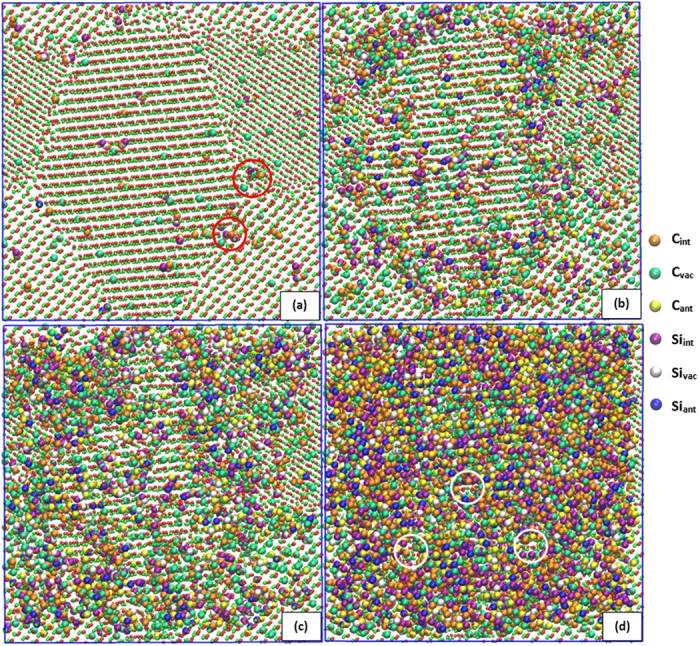
Atomic structures of the amorphization process in nc-SiC. Cross sections of nc-SiC with a width of 0.87 nm are exhibited at 0.01 dpa (**a**), 0.10 dpa (**b**), 0.20 dpa (**c**) and 0.55 dpa (**d**), where the small red spheres represent C atoms and the small green spheres represent Si atoms. The defect type is distinguished by color scale. The legend indicates the six possible defect types: C interstitial (C_int_), Si interstitial (Si_int_), C vacancy (C_vac_), Si vacancy (Si_vac_), C antisite (C_Si_), and Si antisite (Si_C_).

In general, the thickness of the grain boundaries is about 1 nm, but larger disordered regions exist at the triple junctions, which may be extended to 2 nm. Fig. 1(a) shows the atomic cross section of the nc-SiC at a low dose of 0.01 dpa, together with the distribution of defects. The results show that the dominant defects are single interstitials and vacancies at low doses, similar to those found in a single cascade^7^,^8^, but few small defect clusters are created, which contain two or three defects and directly formed by the displacements of C, as circled in Fig. 1 (a). In the present study, the majorities of C and Si interstitials are C-C or C–Si dumbbells, which is consistent with previous simulations^9^,^10^,^11^, and mainly distribute along the GBs. These defects can be formed when a C atom is displaced near a C/Si atom or a pre-existing antisite defect. The nearest-neighbor and next-nearest-neighbor jumps are two main mechanisms for C interstitials to migrate through the C-C and Si-C dumbbells, particularly near the GBs. These short-range diffusions of C interstitials provide possible pathways for them to be absorbed by the GBs. The long-range migrations of defects are not observed at the temperature considered due to the high migration energies of defects in SiC^12^. However, it is observed that the C interstitials near the GB can easily migrate into and also along the GBs, thus forming defect complexes in the GBs. In contrast, C or Si vacancy preferentially stays at the GBs as an individual vacancy. Moreover vacancies located within the grain are essentially immobile and the accumulation of vacancies at the GBs is unfavorable.

The accumulation of defect clusters mainly occurs along the GBs. During continued C displacements, the small clusters coalesce and grow into larger clusters, particularly for the doses ranged from 0.01–0.10 dpa, some of which are associated with the GBs to form amorphous domains, as illustrated in Fig. 1(b,c). We also found that the rate of defect-cluster nucleation along the GBs is larger than that within the grains at low dose levels. It is well known that GBs act as sinks for point defects and increase the annihilation rate of point defects. Therefore, the competition between generation and annihilation of small clusters represents a dynamic process forming defect clusters during the amorphization process, particularly along the GBs. A fully amorphous state is reached at a dose of about 0.55 dpa, as shown in Fig. 1(d). At this dose, the long-range order is completely lost, but there exists a certain degree of local order with partially correlated tetrahedral networks, as circled in Fig. 1(d). The evolution of an atomic slice of nc-SiC with the thickness of 0.87 nm is also included in the Supplemental Materials as Video S1 and S2, showing the different orientations.

Actually the ordered atomic arrangements within the grain and the damage accumulation along the GBs have been already observed by experiments^5^. After electron irradiation with a 300 keV electron beam at 300 K, Ishimaru *et al.*^5^ found that the radiation-induced point defects are rapidly annihilated by the presence of the planar defects and the atomic arrangements remained ordered within the grain. However, the GB is highly damaged, where amorphization occurs. As a result, the shape of grains changed and the nano-columns became narrow under irradiation. The amorphization processes of our present simulations are in good agreement with these experimental observations, and consistent with the molecular dynamics (MD) simulations of irradiated nanocrystalline SiC by Niu *et al.*^13^ who found the same trend of damage accumulation processes in irradiation-induced amorphization. Cascade simulations in nanocrystalline SiC have showed that the number of defects produced in a cascade primarily depends on the volume fraction of GB and more defects are produced along the GB due to the lower threshold displacement energies^14^.

**Figure 2:**
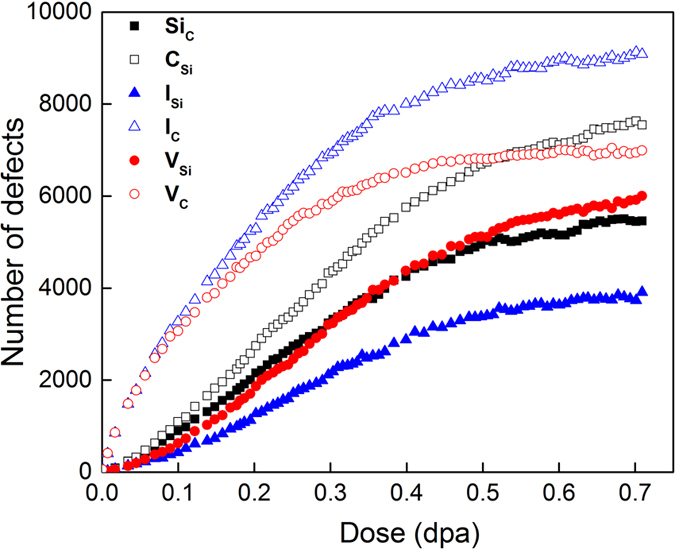
The number of interstitials, vacancies and antisite defects as a function of dose. The Si and C components are presented separately.

To demonstrate the defects production, accumulation and annihilation, the numbers of interstitials, vacancies and antisite defects are shown in Fig. 2 as a function of dose. In general, vacancies, interstitials and antisite defects increase sigmoidally with increasing dose, but C interstitials and vacancies follow a logarithmic function with dose. For C antisites, Si antisites, Si interstitials and Si vacancies, the increases are slow at low dose levels, which may be associated with their low mobilities. However, the number of C interstitials and vacancies significantly increases because of introducing C displacement directly. Due to the presence of the GBs, C displacements result in the increased defect production at the prior-existing defect-disordered regions and the GBs absorb the defect clusters to grow and coalesce. The enhanced clustering due to the GBs leads to a faster increase in interstitial production above a dose of 0.10 dpa, and the increase is very small after 0.55 dpa, which suggests that the nc-a transformation has been achieved. The total number of antisite defects is comparable with that of vacancies, especially at high doses. For example, 12,053 antisite defects are produced as compared with 12,380 vacancies at 0.55 dpa. It should be pointed out that the results in Fig. 2 are obtained using Wigner-Seitz-cell analysis that is based on original lattice sites, as described in the simulation details. Furthermore, it is clear that the number of C interstitials, I_C_, is greater than that of Si interstitials, I_Si_, for all the doses simulated, which is consistent with the smaller threshold energy for C atoms in a SiC system^8^,^15^,^16^.

**Figure 3:**
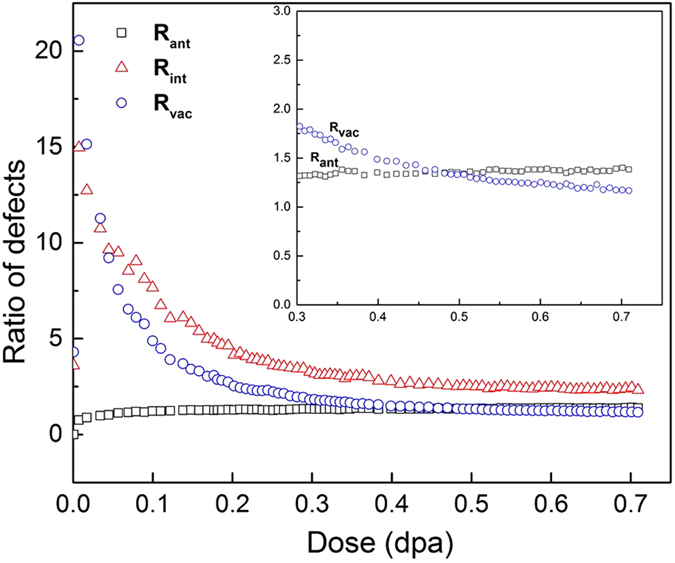
The ratios of various defects. The ratio of the number of C to Si vacancies, R_vac_, is shown as a function of dose, together with those of C to Si interstitials, R_int_, C to Si antisites, R_ant_.

The ratio of the number of C interstitials to Si interstitials, R_int_, as a function of dose is plotted in Fig. 3, together with that of C vacancies to Si vacancies, R_vac_, and C antisites to Si antisites, R_ant_. For three ratios, the saturation values at a dose of 0.55 dpa remain virtually unchanged up to higher dose levels, which suggests that nc-a transformation is achieved under the C displacement condition. The inserted plot shows that the interstitial ratio drops from 15.0 to 2.5 and eventually becomes a constant. The average interstitial ratio at low doses is higher than those found in cascade overlap (3.8^8^) and single cascades (3.5^10^ and 3.2^11^), as well as the final interstitial ratio. The cascade overlap gave a saturate interstitial ratio of 1.7^8^, which is 0.8 lower than our simulation. This may be associated with the fact that only C displacements are introduced in the present simulation, but a cascade in SiC will produce both C and Si interstitials, which gives rise to the low ratio of R_int_ in cascade overlap and a single cascade. Unlike R_int_, R_ant_ logarithmically increases with increasing dose and the increasing range is very small (about 1.40). As shown in the inserted plot, there is a cross over of vacancy and antisite ratios at the dose of 0.48 dpa. After this dose, the large number of C antisite defects is produced and the generation of antisite defects starts playing a key role in the amorphization process. Nevertheless, this plot clearly indicates that the number of C antisite defects is larger than that of Si antisite defects. As seen in Fig. 1, the antisite defects distribute randomly in the nc-SiC. It is of interest to note that the vacancy ratio, R_vac_, sharply increases from 4.3 at the initial C displacement to 20.6 at the dose of 0.01 dpa. As shown in Fig. 2, the number of C vacancies increase faster than Si vacancies. 411 C vacancies are produced as compared with 20 Si vacancies at 0.01 dpa. When the nc-a transformation occurs, the ratio of C to Si vacancies approaches a constant of 1.56 after 0.55 dpa. When a C atom is displaced from its original lattice site, one vacancy defect is created. But Si vacancies are generated by Si displacements induced by C interstitials. This clearly demonstrates that the formation of C-Si or C-C dumbbells and the accumulation of C interstitial defects are able to provide the driving force for the nc-a transformation.

**Figure 4:**
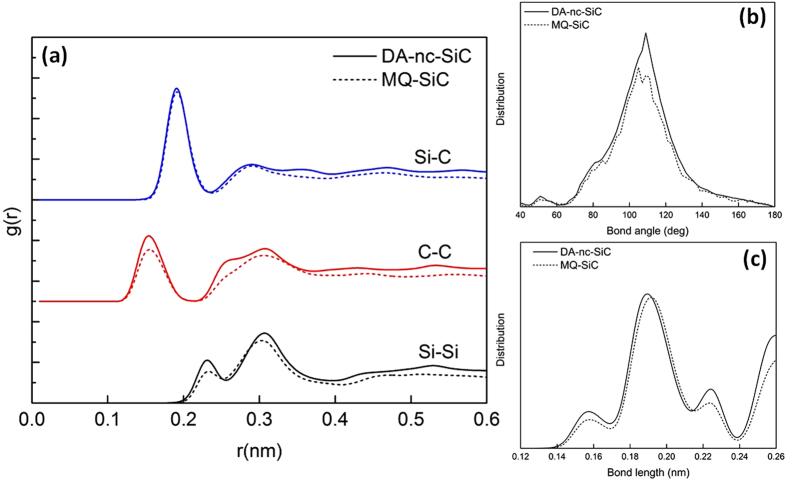
Topological feature of amorphous nc-SiC. (**a**) Calculated pair correlation function, g(r), for the DA-nc-SiC, together with the reference of MQ-SiC sample for comparison. Separate contributions from Si-C, C-C, and Si-Si pairs are shown, respectively. The g(r) of GBs is similar to that of the bulk amorphous sample without long-range order and shows liquid-like structures. (**b**) The bond-angle distribution for DA-nc-SiC and MQ-SiC is compared. (**c**) The bond-length distribution for DA-nc-SiC and MQ-SiC is compared.

The amorphous character of the final structure at 0.55 dpa is illustrated by the calculated pair-correlation function, which is shown in Fig. 4(a), along with that obtained from the melt-quenched (MQ) amorphous sample, where the contributions from Si-C, C-C, and Si-Si pairs are presented separately. The pair-correlation function for displacement amorphized nanocrystalline SiC (DA-nc-SiC) is very similar to that of the MQ-SiC sample, particularly in the positions and size of peaks, and their distributions show the typical systems without periodicity and liquid-like structures. The small peak of C-C pair appears at about 0.15 nm, which is comparable to the nearest-neighbor distance for graphite (0.142 nm) and diamond (0.154 nm). This peak might belong to C-C-C configurations and arise from C π-π interaction. A small peak of C-C pair is at about 0.31 nm corresponding to the different bonding of C-C pairs with Si atoms, such as C-C-Si configurations. It is also noticed that there exists a small shoulder at 0.25 nm between the first and second peaks on the C-C pair, which is very close to the second nearest-neighbor distance of diamond (0.252 nm). The peaks of C-C pairs exhibit the formation of the diamond-like structure of C atoms in the nc-SiC. These different bonds formed by C-C pairs represent sp^2^- and sp^3^-hybridized crystal structures, which have been observed experiments and calculations in an amorphous SiC^17^,^18^. The high peak of the Si-C bonding appears at 0.19 nm corresponding to the nearest-neighbor distance of 3C-SiC, which may be related to C-Si-C or Si-C-Si configurations. The three curves have a common peak near 0.30 nm, which describes the different atomic arrangements of the two species. These results clearly indicate that various bonds could coexist in an amorphous SiC, particularly the formation of C-C and Si-Si bonds, but a certain number of Si-C bonds still remains, which implies that the disordering of SiC does not lead to a fully connected disordered tetrahedral network. From the atomic configuration at the dose of 0.55 dpa (see Fig. 1(d)), there is still a certain degree of atomic arrangements of Si-C tetrahedral network. The bond-angle and bond-length distributions for AD-nc-SiC and MQ-SiC are compared in Fig. 4(b,c). Although the number of atoms for the wide range of bond angle between 60° and 140° are slightly different, the curves share very similar behaviors. The distribution of bond length is also wider than the ordered SiC (see Supplementary Figure S1). These topological analyses indicate that the atomic arrangement of Si and C atoms is completely distorted and a fully amorphous state is achieved.

To identify the distribution and arrangement of C or Si atoms, the total and partial coordination numbers for the DA-nc-SiC are calculated by integrating the coordination number within the first peak before the first minimum in the total pair-correlation function. The coordination numbers for the DA-nc-SiC are listed in Table 1, together with those for a perfect crystal and the MQ-SiC sample. Although DA-nc-SiC and MQ-SiC samples are proceeded by two completely different methods (i.e., displacement-induced amorphization and melting-quenching process), the total coordination numbers (n_total_) are very similar with the values of 3.90 and 3.62 for the DA-nc-SiC and MQ-SiC samples, respectively. The partial coordination number of C (n_C_) is composed of n_CC_ and n_CSi_, which are calculated as 1.04 and 2.87 for the DA-nc-SiC, together with the results 0.92 and 2.77 for MQ-SiC sample. A total C coordination number of 3.91 and 3.69 are also similar. It is noticed that about 25–30% of bonds formed by C atoms are homonuclear for C displacement-induced amorphous nc-SiC. The total C coordination of amorphous SiC smaller than 4.0 has also been predicted by previous calculations^8^,^18^.

## Discussion

GBs can enhance defects to grow and coalesce into clusters to form amorphous domains, which plays an important role in amorphization of nc-SiC. Despite of the existence of GBs sink, nc-SiC can be amorphized by C displacements with a relative high amorphization dose of 0.55 dpa. The competition between production and annihilation of defects represented a dynamic process of the amorphization. Furthermore, the defect production, accumulation and annihilation demonstrate that the driving force for the nc-a transformation is due to the formation and accumulation of C-Si or C-C dumbbells. Therefore, GB enhanced point defect annihilation is proposed to account for the outstanding self-healing behavior of nc-SiC. Under the C displacement condition, Devanathan *et al.*^19^ performed the MD simulation and referred that the amorphization of single crystal SiC occurred by C displacements at the dose of 0.2 dpa at 100 K. Experimentally, Zhang *et al.*^20^ studied defect production and damage accumulation in both single crystal SiC and nano-engineered (NE) SiC containing high-densities of stacking faults at 300 K. The crystalline structure of the NE-SiC achieved complete amorphization at a high radiation dose of about 3 dpa. This value is much larger than that for amorphization of a single crystalline SiC (~0.29 dpa) under similar irradiation conditions. However, some experiments^6^,^21^ on nanocrystalline 3C-SiC with the average nanoparticle sizes of 2.0–4.6 nm suggested that full amorphizations of 3.8 and 4.6 nm grains were achieved at a dose of 0.24 dpa and the minimum dose of ~0.5 dpa at room temperature, respectively. The doses of 0.11 and 0.48 dpa were required for Si-ion-induced amorphizations of 2.0 and 3.0 nm sizes at 400 K, respectively^21^. These results show that the effect of temperature and grain size on amorphization dose is obvious. High temperature will significantly enhance defects to recombine near the GBs and cause the self-healing. Defect accumulation in nc-SiC also can be suppressed by defect recovery via interfaces and GBs.

Under the C displacement condition, nc-SiC can be amorphized with a relative high amorphization dose of 0.55 dpa. The competition between production and annihilation of defects represents a dynamic process of the amorphization. Furthermore, the driving force for the nc-a transformation is due to the formation and accumulation of C-Si or C-C dumbbells at GBs. Therefore, GB-enhanced point defect annihilation is proposed to account for the outstanding self-healing behavior of nc-SiC. Further studies are needed to probe the fundamental radiation damage processes in nc-SiC at long time-scale and high temperatures by performing Monte Carlo simulation combined with MD approach.

## Methods

nc-SiC simulation cells are constructed with a widely used Voronoi cell method by distributing grain centers uniformly in a crystalline 3C-SiC, which is believed to contain the essential geometry of nc materials. A set of grains with random orientations is constructed in a simulation box consisting of 20*a*_0_ × 20*a*_0_ × 20*a*_0_ (*a*_0_ is the lattice constant, 0.436 nm) unit cells (63,280 atoms) to form a nc-SiC sample, corresponding to 4 grains with a mean grain size of 5 nm. The initial configuration is obtained by performing energy optimization for a period of 50 ps at 500 K, then quenching to 0 K for 30 ps, and relaxing the system at 50 K for additional 40 ps. Three dimensional periodic boundary conditions are imposed to model a bulk material.

The MD simulations were carried out using the Moldy code^8^ with a NVT ensemble (constant temperature and constant volume). The temperature in MD simulation is 50 K. We simulated C displacement events by randomly selecting a C atom^19^, moving it a distance ranged from 0.8*a*_0_ to *a*_0_ which exceeds the spontaneous recombination distance for C Frenkel pairs^11^, in a random direction, and then relaxing the cell for 0.40 ps. The process of C atom selection, displacement, and equilibration was repeated until the desired displacement dose was achieved. The present work measures the dose in terms of displacement per atom defined here as the ratio of the number of C displacements introduced to the total number of atoms in the cell. A Wigner-Seitz cell method^22^ is employed to determine the interstitials, vacancies and antisite defects by analyzing atom positions with respect to Voronoy polyhedral centered on ideal lattice sites. A lattice site with an empty Wigner-Seitz cell was recognized as a vacancy, a cell with multiple atoms is an interstitial and a site occupied by a wrong atom type is designated as an antisite defect. Nordlund *et al.*^22^ compared the amount of damage analyzed by different criteria. The results suggested that the Wigner-Seitz method applied on amorphous zones does have the advantage that it immediately tells whether the pocket has an excess or deficiency of atoms compared to an undisturbed lattice. The interactions between atoms were described using Tersoff potentials along with a modification of short-range interactions based on *ab initio* calculations^10^.

In order to characterize the topologies of partial and complete amorphization irradiated in SiC, a reference amorphous sample has been prepared using the same Tersoff potentials. A crystal containing 1,728 atoms was initially heated to 6,000 K, and was further equilibrated for 200 ps to allow proper atomic mixing, and then quenched to 0 K for 20 ps. Although this may not reproduce all the properties of amorphous SiC, it provides a simple method that is sufficient for evaluating the possible aspects of topological features of SiC.

## Additional Information

**How to cite this article**: Chen, D. *et al.* Grain boundary resistance to amorphization of nanocrystalline silicon carbide. *Sci. Rep.* **5**, 16602; doi: 10.1038/srep16602 (2015).

## Supplementary Material

Supplementary Information

Supporting online Video S1

Supporting online Video S2

## Figures and Tables

**Table 1:** Total and partial coordination numbers.

Coordination numbers	Perfect crystal	MQ sample	DA-nc-SiC
n_total_	4.00	3.62	3.90
n_C_	4.00	3.69	3.91
n_CC_	0.00	0.92	1.04
n_CSi_	4.00	2.77	2.87
n_Si_	4.00	3.55	3.90
n_SiSi_	0.00	0.78	1.03
n_SiC_	4.00	2.77	2.87

The total and partial coordination numbers for perfect crystal, MQ amorphous sample, and DA-nc-SiC are listed separately.
