# Supplementary material for: Grain boundary resistance to amorphization of nanocrystalline silicon carbide
Source: Sci Rep. 2015 Nov 12;5:16602. doi: 10.1038/srep16602 (PMC4642319; doi:10.1038/srep16602)
Supplement: Supplementary Information [file srep16602-s1.pdf]

Supplementary Information for

Grain boundary resistance to amorphization of nanocrystalline silicon carbide

Dong Chen[1*], Fei Gao[2*], Bo Liu[1]

[1]*Department of Physics and Electronics, Henan University, Kaifeng 475004, P. R. China*
[2]*Department of Nuclear Engineering and Radiological Sciences, University of Michigan, Ann Arbor, Michigan 48109, USA*

[*]Corresponding author. E-mail: gaofeium@umich.edu (F.G.), dongchen@henu.edu.cn (D.C.)

Table of Contents

1. **Supporting online Video S1:** Animation of an atomic slice of nc-SiC with the thickness of 0.87 nm. The small red and green spheres represent C and Si atoms, respectively.

2. **Supporting online Video S2:** Computational images showing the evolution of an atomic slice of nc-SiC for doses up to 0.71 dpa. Carbon atoms are randomly displaced during the amorphous process.

3. **Supporting online Figure S1:** The distribution of bond length for an ordered SiC. From this plot, the high peak of bond length for the ordered SiC appears at 0.19 nm, which corresponds to the nearest-neighbor distance of 3C-SiC. The distribution of bond length for the ordered SiC is much narrower than the amorphous structure.

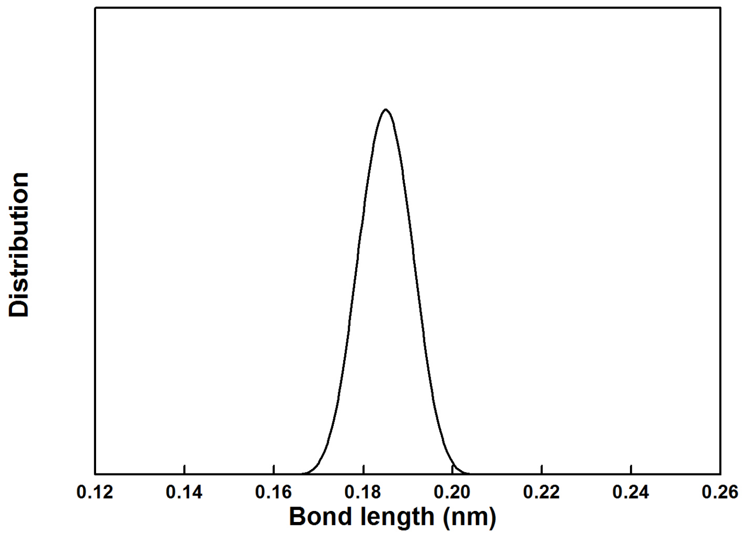

Figure S1 The distribution of bond length for an ordered SiC.
